# Supplementary material for: Quantifying soil accumulation of atmospheric mercury using fallout radionuclide chronometry
Source: Nat Commun. 2024 Jun 26;15:5430. doi: 10.1038/s41467-024-49789-7 (PMC11208417; doi:10.1038/s41467-024-49789-7)
Supplement: Supplementary file 1 — Supplementary Information [file 41467_2024_49789_MOESM1_ESM.pdf]

Supporting Information
for

Quantifying soil accumulation of atmospheric mercury using fallout radionuclide chronometry

Joshua D. Landis, Daniel Obrist, Jun Zhou, et al.

Supporting Information consists of five pages including Figures S1-S4 and Tables S1-S2.

S1. ***Identifying robust soil Hg chronologies***. Soils are notoriously complex, with pedogenic processes redistributing mass vertically and horizontally, through both physical and chemical processes, operating variously at different depths in different horizons, and possibly changing over decadal to centennial timescales with climate, vegetation, and land use disturbances. To every extent possible we sampled putative 'reference' soils at sites of known land-use histories, undisturbed for >150 years, on flat ground, near local topographic high points or ridgetops to reduce the influence of horizontal flowpaths, and over relatively large areas (30 x 30 cm) to reduce localized heterogeneities. The gold standard of ensuring that a soil site may be trusted to produce an honest record of atmospheric deposition is the concordance of multiple independent chronometers. The LRC age model typically dates the ^{241}Am bomb-pulse to its expected peak of 1963-4 with errors <5 years or +10%. Here our global compilation has geometric mean =1965.8 ±1.9 SE, *k*=1, *n*=18; Table S1). When this is the case, total soil ^{210}Pb and ^{241}Am inventories are tightly correlated based on their common atmospheric source (R^2=0.88, *n*=19; Fig. S1). When this is not the case, the ^{210}Pb and ^{241}Am have likely been redistributed differentially with respect to each other by some of the disturbance problems listed above, and due to the different timeframes over which they are deposited. ^{210}Pb is deposited continuously and forms an exponential depth profile, whereas ^{241}Am was deposited in a pulse and appears as a sharp peak in the soil subsurface at depths ranging 5-10 cm.

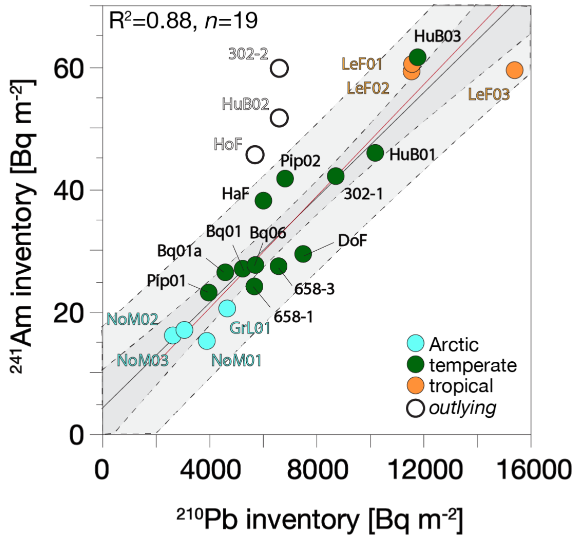

Figure S1: Linear correlation of ^{241}Am and ^{210}Pb soil inventories for Arctic, temperate/boreal and tropical sites. Two-sigma confidence intervals for fit and prediction are shaded. Typical ^{210}Pb:^{241}Am = 233 +9 (1-sigma SD). Points colored white are considered outlying and possibly impacted by disturbance.

Soils that do not produce accurate ^{241}Am dates, fall outside the Pb-Am correlation confidence intervals (Fig. S1), or have total Hg inventories significantly different than ecosystem means (Table S1), are omitted from analysis of long-term Hg chronologies. For example, in reconstructing Hg accumulation for northeastern soils, we omitted 3 pits likely to have been disturbed (302-2, 658-1, and HoF01), and two that show atypical Hg low-accumulation as described in the main text (658-3 and DoF01). Historical reconstruction with sites omitted in the main text are shown in the alternative Fig. S2 below. Concentrations of Hg for all soil pits are shown in Fig. S3.

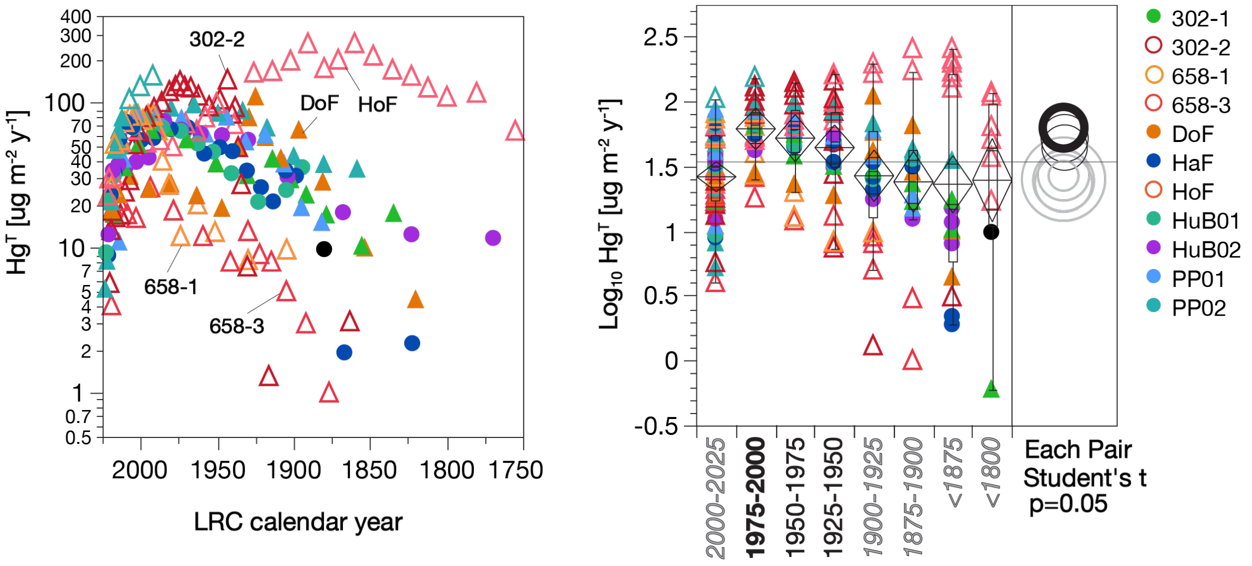

Figure S2. Reconstructed rates of soil Hg accumulation, including outlying sites with evidence of disturbance (HoF, 302-2) or Hg remobilization (658-1, 658-3).

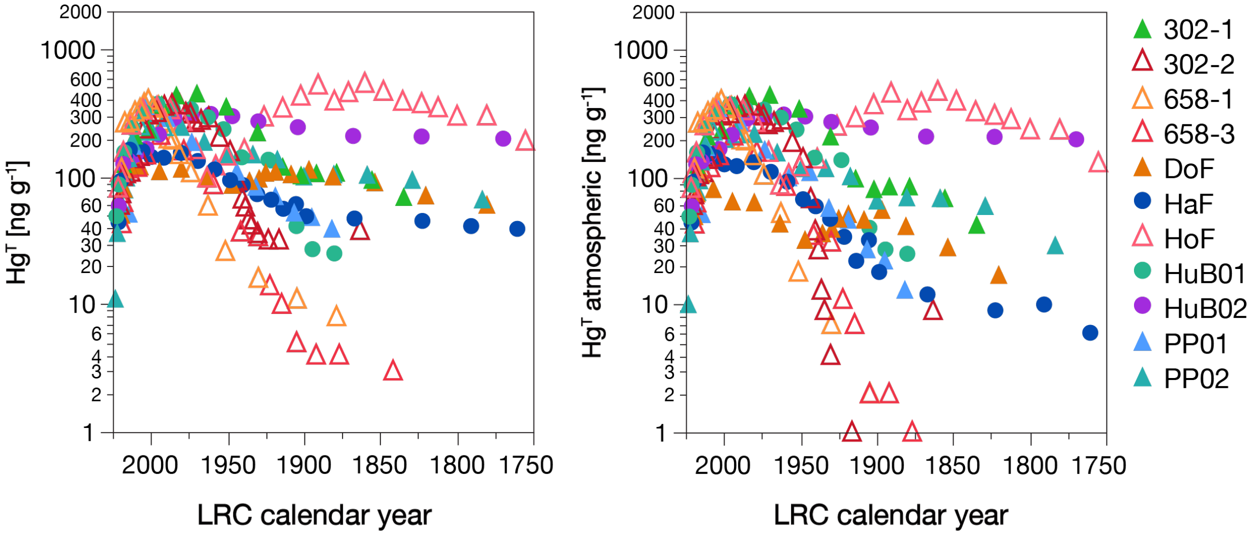

Figure S3. Total Hg^T and atmospheric Hg^T concentrations in temperate soils versus calendar year. Atmospheric Hg^T has removed estimated geogenic contributions by normalization with Al.

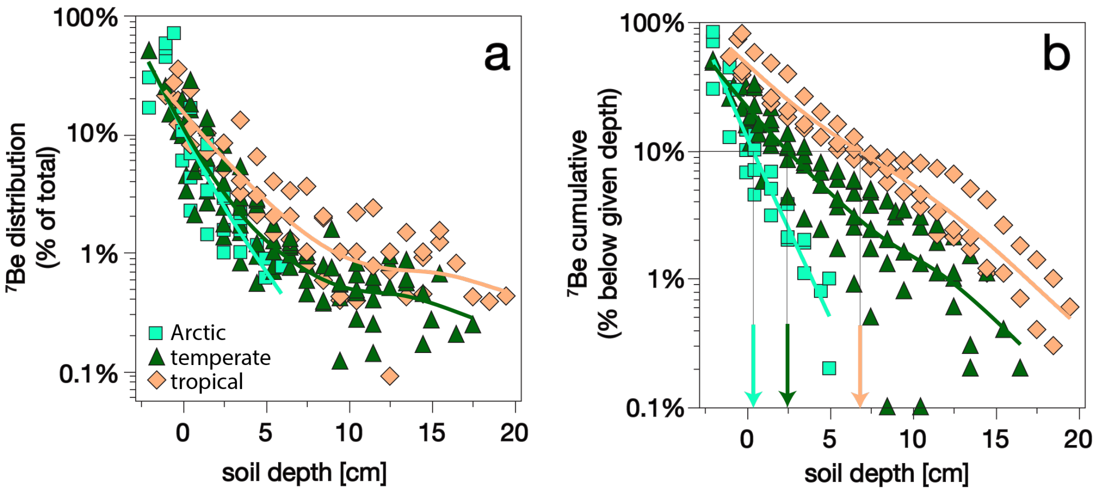

Figure S4: (a) mass-depth distributions of ^{7}Be (half-life 54 days) in global soils. Lines show spline best fits. (b) cumulative mass distributions for ^{7}Be below a given depth. In Arctic soils 10% of ^{7}Be is found below soil depths of 0.5 cm, and corresponding distributions in temperate and tropical soils are 2.5 cm and 7 cm, respectively.

Table S1: soil layer ages, Hg concentrations, inventories, and fluxes

	coniferous			*deciduous*			*evergreen*			*tundra*		
horizon	years	σ	*n*	years	σ	*n*	years	σ	*n*	years	σ	*n*
foliage	1.7	0.3	7	0.43	0.23	1	0.71	0.28	3	4	1	10
Oi	3	0	11	2	0	10	1	0	4	15	3	7
Oe	10	1	30	5	1	13	4	1	7	33	6	8
Oa	28	2	32	34	5	26	10	3	9	78	12	12
A	74	6	33	72	21	5	18	3	26	139	31	5
E	91	11	15	139	44	4						
B	118	10	31				43	6	39	160	44	3

	coniferous			*deciduous*			*evergreen*			*tundra*		
horizon	ng g^{-1}	σ	*n*	ng g^{-1}	σ	*n*	ng g^{-1}	σ	*n*	ng g^{-1}	σ	*n*
foliage	49	16	5	22	4	3	23	6	2	35	5	9
Oi	72	18	10	53	6	9	54	10	4	106	18	6
Oe	143	22	30	113	12	11	116	17	7	146	23	7
Oa	270	39	32	206	14	32	131	17	9	126	16	11
A	121	17	34	79	12	6	110	8	26	61	9	9
E	28	6	16	29	4	6						
B	79	9	57	62	6	14	78	5	42	11	2	8

	coniferous			*deciduous*			*evergreen*			*tundra*		
horizon	µg m^{-2}	σ	*n*	µg m^{-2}	σ	*n*	µg m^{-2}	σ	*n*	µg m^{-2}	σ	*n*
foliage	18	6	5	7	3	1	8	3	2	12	3	9
Oi	36	9	10	25	5	8	14	4	4	99	26	6
Oe	120	18	30	81	13	11	104	24	7	214	52	7
Oa	408	60	32	474	46	32	169	35	9	360	72	11
A	808	117	33	590	124	6	378	48	26	621	136	9
E	240	48	16	261	55	6						
B	793	89	57	458	65	14	931	93	43	158	36	8

	coniferous			*deciduous*			*evergreen*			*tundra*		
horizon	µg m^{-2} y^{-1}	σ	*n*	µg m^{-2} y^{-1}	σ	*n*	µg m^{-2} y^{-1}	σ	*n*	µg m^{-2} y^{-1}	σ	*n*
foliage	8.8	2.8	6	13.9	4.8	1	20.1	6.2	4	3.3	0.5	9
Oi	18.2	4.7	10	13.9	1.9	8	17.2	5.3	4	8.9	1.8	6
Oe	29.6	4.7	30	26.6	3.2	11	70.4	17.1	7	12.8	2.4	7
Oa	57.0	8.7	32	47.2	3.8	26	105.0	22.8	9	11.7	1.8	11
A	55.5	8.4	33	47.3	8.2	5	108.5	14.6	26	15.3	3.3	5
E	18.5	4.1	14	20.7	4.5	3						
B	39.8	6.5	28				100.4	11.4	37	11.1	3.0	3

Table S2: site locations and characteristics for reconstruction of Hg accumulation in soils by FRN chronometry

site	town	region	site	pit code[a]	canopy species	biome	ecotype	elevation m a.s.l.	lat N	long W	MAT C	MAP cm	soil order	parent lithology	experimental condition	Hg^T mg m^{-2}	[b]Hg^T_{atm} mg m^{-2}	$^{210}Pb_{xs}$ Bq m^{-2}	^{241}Am Bq m^{-2}	*bomb year***	^{137}Cs Bq m^{-2}	$^{210}Pb/Am$ R	$^{210}Pb/Hg$ R
Beaver Meadow	Sharon	VT	BeM	FLH	*Pinus strobus*	*temperate*	*coniferous*	464	43.80	72.40	6.8	108	inceptisol	schist, phyllite									
				FLH	*Quercus rubra*	*temperate*	*deciduous*	464	43.80	72.40			inceptisol	schist, phyllite									
Camel's Hump State Forest	Duxbury	VT	CaH	FLH	*Sphagnum*	*temperate*	moss	1067	44.32	72.88	2.4	203	spodosol	schist, phyllite									
Downer State Forest	Strafford	VT	DoF	FLH	*Pinus resinosa*	*temperate*	*coniferous*	460	43.80	72.39	6.8	108	inceptisol	schist, phyllite									
				FLH	*Tsuga canadensis*	*temperate*	*coniferous*	460	43.80	72.39			inceptisol	schist, phyllite									
				DoF01	*Pinus strobus*	*temperate*	*coniferous*						inceptisol	schist, phyllite	reference	13.9	6.1	7529	29.3	*1963*	1689	257	1232
Experimental Lakes Area	Kenora	ON	ELA302	**302-1**	*Picea mariana*	*boreal*	*coniferous*	460	49.68	93.77	3.5	80	inceptisol	gneiss	reference	9.0	8.7	8748	42.0	*1966*	1484	209	1004
				302-2	*Picea mariana*	*boreal*	*coniferous*						inceptisol	gneiss	reference	10.0	7.6	6638	59.6	*1967*	1217	111	872
				658-1	*Picea mariana*	*boreal*	*coniferous*						inceptisol	gneiss	disturbed, fire	3.9	3.2	5707	24.0	*n/a*	842	237	1763
				658-3	*Picea mariana*	*boreal*	*coniferous*						inceptisol	gneiss	reference	1.9	1.6	6611	27.3	*1966*	1376	243	4161
Harvard Experimental Forest	Petersham	MA	HaF	FLH	*Quercus rubra*	*temperate*	*deciduous*	340	42.32	72.11	8.8	122	inceptisol	glacial till									
				**HaF**	*Quercus rubra*	*temperate*	*deciduous*								forest regrowth	8.8	5.8	6044	38.0	*1964*	1139		
Howland Experimental Forest	Howland	ME	HoF	FLH	*Tsuga canadensis*	*temperate*	*coniferous*	60	45.20	68.70	6.2	115	spodosol	granitic till									
				HoF	*Tsuga canadensis*	*temperate*	*coniferous*								woody debris	34.1	31.0	5730	45.5	*1947*	1499 >	126	185
Hubbard Brook Experimental Fores	N. Woodstock	NH	HuB	**HuB01**	*Betula alleghaniensis*	*temperate*	*deciduous*	584	43.96	71.72	6.7	150	spodosol	granitiic till	hillslope	5.9	5.8	10223	45.8	*1967*	841 >	223	1748
				**HuB02**	*Betula alleghaniensis*	*temperate*	*deciduous*	410					spodosol	granitiic till	hillslope	8.7	8.7	6636	51.5	*1951*	908 >	129	767
				Hub03	*Picea rubens*	*temperate*	*coniferous*	710					spodosol	granitiic till	hillslope			11807	61.4	*n/a*	4263	192	
Berlin chloralkakli	Berlin	NH	BqP	BqP1	*Betual*	*temperate*	*deciduous*						spodosol	alluvium	Hg pointsource	*n/a*		5270 *	26.9	*n/a*	1700	196	
				BqP1a	*Tsuga canadensis*	*temperate*	*coniferous*	298	44.45	71.18		107	spodosol	alluvium	Hg pointsource	*>28*	*>28*	4619 *	26.3	*n/a*	1101 >	176	
				BqP6	*Tsuga canadensis*	*temperate*	*coniferous*						spodosol	alluvium	Hg pointsource	*n/a*		5747	27.5	*1962*	1301 >	209	
Mink Brook watershed	Etna	NH	MiB	FLH	*Acer saccharum*	*temperate*	*deciduous*	435	43.68	72.18		110	inceptisol	amphibolite									
Shattuck Observatory	Hanover	NH	ShO	timeseries	*Quercus rubra*	*temperate*	*deciduous*	165	43.70	72.29	6.9	109	spodosol	amphibolite									
				**PiP01**	*Pinus strobus*	*temperate*	*coniferous*							alluvial till	reference	10.5	8.1	3999	23.0	*1966*	896 >	174	492
				**PiP02**	*Pinus strobus*	*temperate*	*coniferous*							alluvial till	forest cut	19.2	13.8	6854	41.6	*1957*	1752	165	495
Woody Adams Conservation	Norwich	VT	WoA	FLH	*Quercus rubra*	*temperate*	*deciduous*	425	43.77	72.35	6.8	111	inceptisol	schist, phyllite									
				FLH	*Acer saccharum*	*temperate*	*deciduous*	425	43.77	72.35			inceptisol	schist, phyllite									
				FLH	*Pinus strobus*	*temperate*	*coniferous*	425	43.77	72.35			inceptisol	schist, phyllite									
				FLH	*Tsuga canadensis*	*temperate*	*coniferous*	425	43.77	72.35			inceptisol	schist, phyllite									
Seward Peninsula	Nome	AK	NoM	NoM01	*Sphagnum*	*Arctic*	moss	120	64.98	166.21	-6.0	43	gellisol	phyllite	hillslope	2.8	2.5	3928	15.1	*1965*	925		
				NoM02	*Sphagnum*	*Arctic*	moss						gellisol	phyllite	reference	3.3	2.5	3092	16.9	*1962*	748		
				NoM03	*Sphagnum*	*Arctic*	moss						gellisol	phyllite	hillslope	2.3	2.0	2660	16.0	*1967*	769		
Sisimuit	Sisimuit	GR	GRL	GrL1	*Sphagnum*	*Arctic*	moss						inceptisol	gneiss		2.7	2.5	4690	20.4	*1967*	611		
Luquillo Experimental Forest	Luquillo	PR	LeF	LeF01	*Dacryodes*	*tropical*	*evergreen*	440	18.32	65.82	24.0	260	ultisol	volaniclastic	reference	30.9	13.4	11601	60.3	*1978*	2910		
				LeF02	*Cyrilla*	*tropical*	*evergreen*	773	18.28	65.79	22.0	360	inceptisol	diorite	reference	21.5	11.3	11580	59.1	*1982*	1910		
				LeF03	*Eugenia*	*tropical*	*evergreen*	1078	18.27	65.76	20.0	420	inceptisol, hydric	diorite	reference	13.6	8.6	15432	59.3	*1962*	2058		
															temperate median	9.0	7.6	6637	42	*1965*	1297	192	938
															SD	8.8	7.9	2131	13	*7*	931	51	1140
															CV	98%	104%	32%	31%	*12.5%*	72%	26%	122%
															count					*11.0*		13.0	10.0
															SE					*3.8%*		7.3%	38.4%

**peak loading or cumulative deposition interpolated to 48.3% in 1963 (Appleby 1991)

[a]FLH denotes collection of foliage, litter and humus only; named pits were quantitative to 50 cm or refusal

[b]atmospheric Hg is distinguished from soil total Hg using Al as an index element

*not at steady state

>value is underestimated due to deep percolation

red text highlights outlying values
